# Predictors of Metastatic Lymph Nodes at Preoperative Staging CT in Gastric Adenocarcinoma

**DOI:** 10.3390/tomography8030098

**Published:** 2022-04-22

**Authors:** Filippo Crimì, Quoc Riccardo Bao, Valentina Mari, Chiara Zanon, Giulio Cabrelle, Gaya Spolverato, Salvatore Pucciarelli, Emilio Quaia

**Affiliations:** 1 Institute of Radiology, Department of Medicine-DIMED, University of Padova, 35128 Padova, Italy; filippo.crimi@unipd.it (F.C.); zanon.chiara.9@gmail.com (C.Z.); giulio.cabrelle@gmail.com (G.C.); emilio.quaia@unipd.it (E.Q.); 2 General Surgery 3, Department of Surgical, Oncological and Gastroenterological Sciences-DISCOG, University of Padova, 35128 Padova, Italy; quocriccardo.bao@unipd.it (Q.R.B.); valentina.mari@studenti.unipd.it (V.M.); puc@unipd.it (S.P.)

**Keywords:** CT, gastric cancer, lymph node

## Abstract

Background. The aim of this study was to identify the most accurate computed-tomography (CT) dimensional criteria of loco-regional lymph nodes (LNs) for detecting nodal metastases in gastric cancer (GC) patients. Methods. Staging CTs of surgically resected GC were jointly reviewed by two radiologists, considering only loco-regional LNs with a long axis (LA) ≥ 5 mm. For each nodal group, the short axis (SA), volume and SA/LA ratio of the largest LN, the sum of the SAs of all LNs, and the mean of the SA/LA ratios were plotted in ROC curves, taking the presence/absence of metastases at histopathology for reference. On a per-patient basis, the sums of the SAs of all LNs, and the sums of the SAs, volumes, and SA/LA ratios of the largest LNs in all nodal groups were also plotted, taking the presence/absence of metastatic LNs in each patient for reference. Results. Four hundred and forty-three nodal groups were harvested during surgery from 107 patients with GC, and 173 (39.1%) were metastatic at histopathology. By nodal group, the sum of the SAs showed the best Area Under the Curve (AUC), with a sensitivity/specificity of 62.4/72.6% using Youden’s index with a >8 mm cutoff. In the per-patient analysis, the sum of the SAs of all LNs in the loco-regional nodal groups showed the best AUC with a sensitivity/specificity of 65.6%/83.7%, using Youden’s index with a >39 mm cutoff. Conclusion. In patients with GC, the sum of the SAs of all the LNs at staging CT is the best predictor among dimensional LNs criteria of both metastatic invasion of the nodal group and the presence of metastatic LNs.

## 1. Introduction

Gastric adenocarcinoma is the fifth most frequently diagnosed cancer, and the third cause of cancer-related death. In 2020, over a million new diagnoses of gastric cancer (GC) and 768,000 deaths were estimated worldwide [1]. Surgery remains the only curative treatment for GC, but most patients relapse after surgical resection [2,3,4]. Extended lymph node (LN) dissection improves survival [5], but also increases surgical morbidity and mortality [6,7,8]. A multidisciplinary approach is consequently recommended for locally advanced GC, as perioperative chemotherapy promotes tumor downstaging, and increases progression-free and overall survival (OS) [9]. Adding preoperative radiotherapy may also improve the major pathologic response rate, reduce LN metastases, and improve OS [10,11]. LN metastasis (N stage) is one of the factors most affecting the survival of patients with GC [5,12,13,14,15]. After surgery, patients staged as N0 reportedly had a 5-year OS of 86.1%, which dropped dramatically to 58.1, 23.3, and 5.9%, respectively, in N1, N2, and N3 [13]. Clinical N stage is therefore essential for deciding the appropriate treatment for each patient, such as perioperative chemotherapy and radiotherapy, followed by surgical resection in cases of clinically suspected nodal metastases [16]. The availability of diagnostic tools such as magnetic resonance (MR), CT scanning, and 18F-FDG PET-CT enables accurate disease staging [17]. Moreover, endoscopic ultrasound (EUS) is indicated for assessing T stage and peri-gastric nodal stations [18], but its accuracy is highly operator-dependent, and EUS is not available at all centers [19]. CT is widely used in daily clinical practice for the preoperative staging of patients with GC. Its sensitivity and specificity for detecting LN metastases are in the range of 62.5 to 91.9%, and 50.0 to 87.9%, respectively [17]. The radiological criteria for defining LN metastases are still a matter of debate, however. Previous studies were based on various radiological benchmarks, such as long-axis (LA) or short-axis (SA) diameters, enhancement pattern, or nodal volume [20,21,22,23], but no studies have compared all these parameters. 

The aim of our study was to identify and compare dimensional features of loco-regional metastatic LNs in preoperative CT scans by matching the preoperative radiological and histopathological data for every single LN station, in an effort to establish the best dimensional predictor of LN metastases.

## 2. Materials and Methods

### 2.1. Patient Selection and Definition of Outcomes

This was a retrospective observational study, approved by the local ethics committee (number of protocol 0050680 approved on 10 August 2021). All consecutive patients who underwent curative-intent surgery for GC between 2010 and 2020 were extracted from the prospectively maintained database of our tertiary-level University Hospital. Patients who underwent palliative or non-radical (R2) surgery, those with gastric neoplasms other than adenocarcinoma (e.g., benign, carcinoids, gastrointestinal stromal tumors, etc.), and those with no CT scan available or obtained more than 4 weeks before their surgical procedure were excluded. Demographic and clinicopathological data, and tumor- and therapy-related variables were extracted, including type of surgery, extent of lymphadenectomy (D1 vs. D2), and perioperative treatment (chemotherapy or chemoradiotherapy). Lymphadenectomy was classified as D1 or D2 according to the Japanese Gastric Cancer Association (JGCA) guidelines, 5th edition [24]. Data on postoperative outcome, such as length of hospital stay (LOS), and type of complication according to the Clavien-Dindo classification [25], were included. For each surgical specimen, a dedicated gastrointestinal pathologist reported: site of lesion, tumor size, histological type, grade, total number of LNs harvested, number and sites of metastatic LNs, and stage of disease according to the American Joint Committee on Cancer (AJCC) staging system, 8th edition [26]. LN status was recorded systematically, considering the presence or absence of metastatic nodes for each nodal station, the total number of nodes retrieved in the specimen, the total number of metastatic nodes, and the number of metastatic LNs out of the total number of LNs harvested, or LN ratio (LNR). Pathological margin status was classified as microscopically negative (R0) or positive (R1). All patients underwent a standard oncological follow-up, according to Italian guidelines [27].

### 2.2. Radiological Analysis

All patients underwent a staging CT scan, according to national and international guidelines [16,27], using a 64-slice scanner (Somatom Sensation, Siemens Healthineers, Erlangen, Germany). CT images were acquired before and after intravenous injection of 2 mL/kg of Iohexol 350 mg I/mL (Omnipaque, GE Healthcare, Milwaukee, WI, USA), followed by a 50 mL saline flush. A cranio-caudal image acquisition was used. Technical parameters of CT were the following: 120 kV tube voltage; 300 mAs effective dose; 0.37 sec rotation time; 0.6 mm detector collimation; 0.7 pitch. Slice thickness was 5 mm for unenhanced acquisition and 3 mm for arterial and venous scans. All images analyzed had a soft-tissue reconstruction with a 30B kernel. Arterial scan was acquired 15 s after the achievement of 100 HU within the aortic lumen (bolus tracking technique) and a venous phase of 80 s after contrast administration.

**Figure 1:**
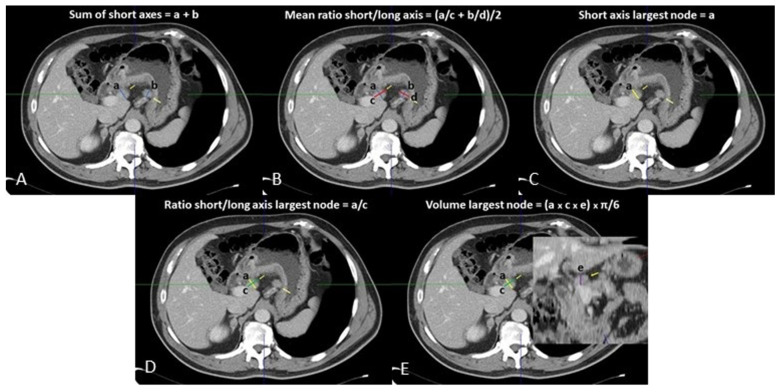
Loco-regional CT measurements: (**A**) sum of short axes; (**B**) mean of ratios of short/long axes; (**C**) short axis of largest lymph node; (**D**) ratio of short/long axis of largest lymph node; (**E**) volume of largest lymph node.

Venous phase images were analyzed on a dedicated workstation (Vitrea2 FX version 6.3, Vital Images, Plymouth, MN, USA). The principal gastric lymphatic stations (paracardial, small and large curve, supra- and sub-pyloric, along left gastric vessels, hepatic artery, celiac trunk, splenic artery, at splenic hilum, retro-pancreatic, and hepato-duodenal) were examined jointly by two abdominal radiologists (with 15 and 5 years of experience), blinded to the pathological diagnosis of the resected LNs. Nodes < 5 mm in maximum size were disregarded. All loco-regional LNs ≥ 5 mm were identified, and their orthogonal axes were measured: the smallest and largest axes on the axial plane were considered as the SA and LA, respectively, while the third measurement obtained was the maximum sagittal axis (Figure 1). The volume of the largest LN per nodal station was calculated according to the formula: SA × LA × sagittal axis × π/6 (mL) (Figure 1).

Five variables per nodal station (the sum of the SAs, the volume, SA, and SA/LA ratio of the largest LN, and the arithmetical average of the SA/LA ratios of all LNs) were recorded in a database. After that, the measurements and the histopathological results of each loco-regional nodal group were reviewed and matched by the two radiologists with the supervision of a dedicated pathologist.

### 2.3. Statistical Analysis

Discrete variables are expressed as means ± standard deviations, or medians and interquartile ranges (IQR), as appropriate. Categorical data are described as absolute numbers and percentages. The SA, volume, and SA/LA ratio of the largest LN, and the sum of the SAs and mean of the SA/LA ratios of all LNs in a given nodal group were compared between metastatic and nonmetastatic LN groups. Continuous variables showing a normal distribution with the Shapiro-Wilk test were analyzed using parametric tests. Nonparametric tests (Mann-Whitney) were used for a skewed distribution. Statistical significance was set at *p* < 0.05. OS and disease-free survival (DFS) were estimated using the Kaplan-Meier method, and the log-rank test were used to compare the curves. Each outcome was calculated from the date of surgery to the date of the event (recurrence, death, or last follow-up). Receiver Operating Characteristic (ROC) curves were plotted to assess the diagnostic accuracy of the different methods (the sum of the SAs of LNs in each nodal group, the volume, SA, and SA/LA ratio of the largest LN in the nodal group, and the arithmetical average of the SA/LA ratios of all LNs), taking for reference the categorical status of metastatic/nonmetastatic definition of the nodal group (presence of at least one metastatic LN in the nodal group at histopathology). Then, ROC curves were plotted to examine the accuracy of the sum of the SAs of all the LNs in all the nodal groups, the sum of the SAs of the largest LNs in all the nodal groups, the sum of the volumes of the largest LNs in all the nodal groups, the mean of the SA/LA ratios of the largest LNs in all the nodal groups, and the mean of the SA/LA ratios of all the LNs in all the nodal groups on a per-patient basis, taking for reference the presence of positive or negative LNs in a given patient (i.e., presence/absence of at least one positive nodal group among those examined from the same patient). MedCalc^TM^ (MedCalc Software Ostend Belgium, vers. 19.7.2) was used for the statistical analysis.

## 3. Results

### 3.1. Patients’ Characteristics

**Figure 2:**
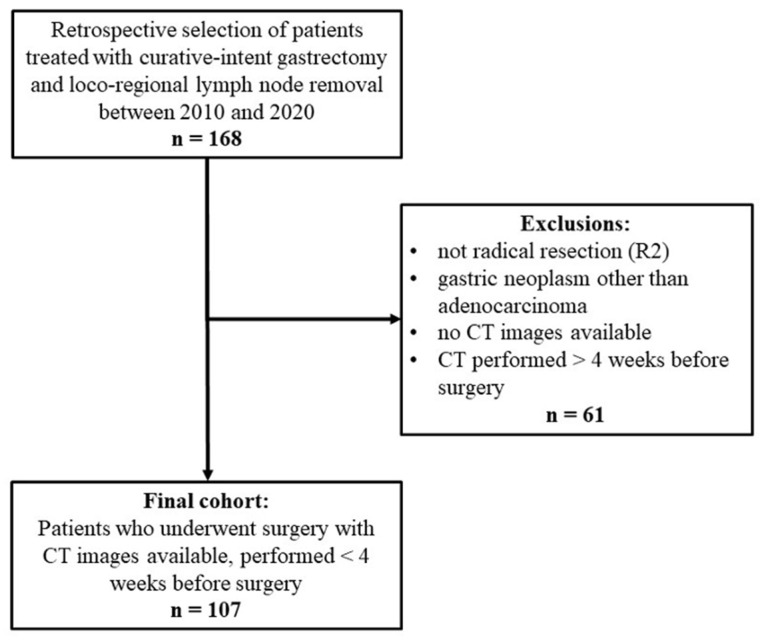
Study flowchart.

In all, 168 patients were treated surgically with curative intent between 2010 and 2020. Sixty-one were excluded for the purposes of our study, and 107 were enrolled, as shown in the flow-chart (Figure 2). Table 1 shows the patients’ demographic and clinical characteristics. At latest follow-up, 33 (30.8%) patients had experienced a recurrence and 58 (54.2%) had died. Patients’ pathological features are summarized in Table 2. 

A total of 443 nodal groups were harvested during surgery and subsequently underwent histopathological analysis: 173 (39.1%) proved to be metastatic, with 69 patients (64.5%) revealing at least one metastatic nodal group (pN+).

### 3.2. Radiological Results

The SA and volume of the largest LN in each nodal group showed a statistically significant difference between the metastatic and nonmetastatic groups (7 (6–9) vs. 6 (5–7) mm, *p* < 0.0001; 280.8 (174.7–624.0) mm^3^ vs. 195.8 (130.0–305.7) mm^3^, *p* < 0.0001), as did the sum of the SAs (11 (7–17) vs. 6 (5–10) mm, *p* < 0.0001), while the SA/LA ratio of the largest LN in a given nodal group, and the mean of the SA/LA ratios of all the LNs in the nodal group, showed no significant difference between the two groups (Table 3).

### 3.3. Analysis by Nodal Group

**Figure 3:**
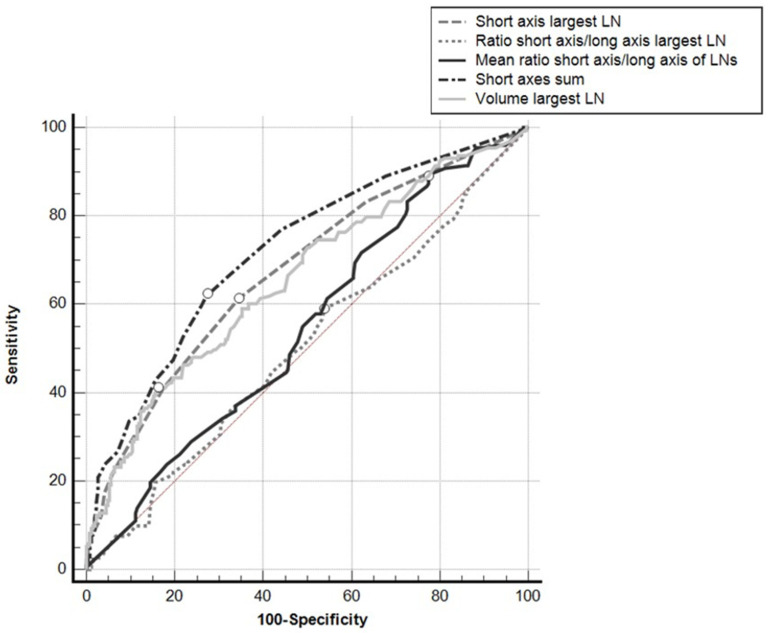
ROC curves for the different parameters used, taking positive/negative results for the nodal groups at histopathology for reference. White dots: Youden’s indexes.

ROC curve analysis showed that the sum of the SAs obtained the best Area Under the Curve (AUC) (0.721; 95%CI: 0.677–0.762), which was significantly greater than the areas obtained from: the SA of the largest LN in a given nodal group (0.675; 95%CI: 0.630–0.719; *p* = 0.0192); the volume of the largest LN in the nodal group (0.657; 95%CI: 0.611–0.701; *p* = 0.0029); the SA/LA ratio of the largest LN in the nodal group (0.504; 95%CI: 0.456–0.551; *p* < 0.0001); the mean of the SA/LA ratios of the LNs in the nodal group (0.544; 95%CI: 0.496–0.591; *p* < 0.0001) (Figure 3).

The AUCs of the ROC curves for the mean of the SA/LA ratios of all the LNs in the nodal group and for the SA/LA ratio of the largest LN in the nodal group did not differ significantly from the area under the identity line (*p* = 0.092 and *p* = 0.893, respectively), while the AUCs for the other parameters did differ significantly from the area under the identity line (*p* < 0.001). 

Using Youden’s index, a cutoff of >8 mm for the sum of the SAs of all the LNs in a given nodal group showed a sensitivity of 62.4% and a specificity of 72.6%; a cutoff of >6 mm for the SA of the largest LN showed a sensitivity of 61.3% and a specificity of 65.6%; for the volume of the largest LN a cutoff of >378.6 mm^3^ obtained a sensitivity of 41.0% and a specificity of 83.7%.

### 3.4. Patient-Based Analysis

**Figure 4:**
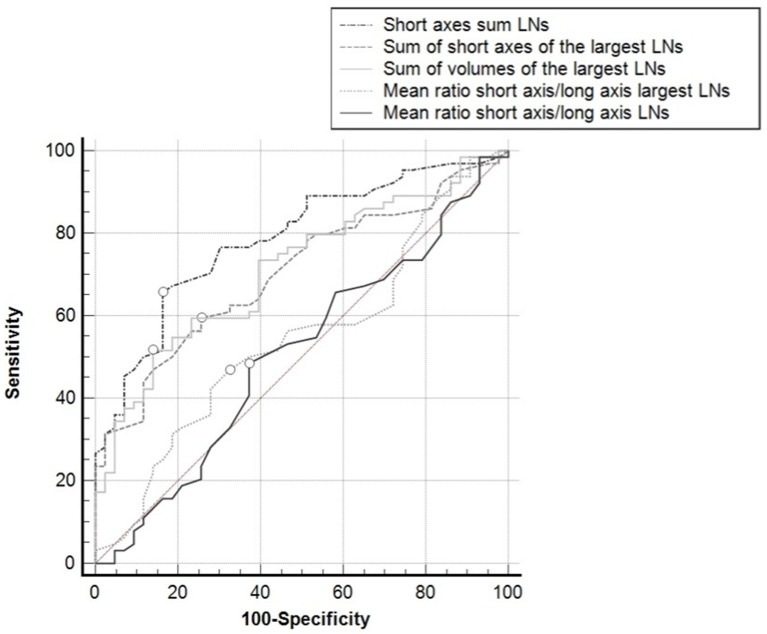
ROC curves for the different parameters used, taking the positive/negative classification of the patients for reference (i.e., presence of at least one metastatic lymph node in one loco-regional nodal group at histopathology). White dots: Youden’s indexes.

Grouping the results on a per-patient basis, the sum of the SAs of all the LNs in all the nodal groups showed the best AUC (0.787; 95%CI: 0.697–0.860), which was significantly greater than the area obtained from the sum of the SAs of the largest LNs in all the nodal groups (0.703; 95%CI: 0.607–0.788; *p* = 0.0016), the sum of the volumes of the largest LNs in all the nodal groups (0.657; 95%CI: 0.611–0.701; *p* = 0.0060), the mean of the SA/LA ratios of the largest LNs in all the nodal groups (0.539; 95%CI: 0.440–0.636; *p* = 0.0002), or the mean of the SA/LA ratios of all the LNs in all the nodal groups (0.508; 95%CI: 0.410–0.606; *p* = 0.0005) (Figure 4).

The AUC of all the ROC curves differed significantly from the identity line (*p* < 0.001), except for the mean of the SA/LA ratios of the largest LNs in the nodal groups (*p* = 0.4932), and the mean of the SA/LA ratios of all the LNs in all the nodal groups (*p* = 0.890). Using Youden’s index, a >39 mm cutoff for the sum of the SAs of the LNs in all the nodal groups showed a sensitivity of 65.6% and a specificity of 83.7%; a >28 mm cutoff for the sum of the SAs of the largest LNs in all the nodal groups showed a sensitivity of 59.4% and a specificity of 74.4%; for the sum of the volumes of the largest LNs in all the nodal groups, a cutoff of >1498 mm^3^ achieved a sensitivity of 51.6% and a specificity of 86.0%.

## 4. Discussion

Accurate staging to identify advanced disease is essential in GC, as patients with metastatic LNs at preoperative staging can benefit from chemo- and/or radiotherapy before surgery [9,11,28,29]. CT has already proved an accurate and feasible method for detecting LN metastases in GC [17,30,31]. Many different features have been investigated as radiological markers of metastatic LNs, such as short- or long-axis diameters [22,32,33], or the sum of LN diameters [21], attenuation greater than 100 HU in the postcontrast portal venous phase, a round shape [23], and iodine concentration in the arterial and venous phases [34]. We chose to consider and compare several parameters that are easy to measure and objectify: the SA of the largest LN; the SA/LA ratio of the largest LN; the mean of the SA/LA ratios of all LNs in a nodal group; the sum of the SAs of all LNs in the nodal group; and the volume of the largest LN. In our analysis by nodal group, the sum of the SAs of all LNs in the group showed the best accuracy, obtaining the greatest AUC on ROC analysis (0.721; 95%CI 0.677–0.762). The difference was statistically significant vis-à-vis the SA of the largest LN, the volume of the largest LN, the SA/LA ratio of the largest LN, or the mean of the SA/LA ratios of all the LNs in the nodal group. With a cut off of >8 mm, the sum of the SAs showed the best sensitivity and specificity, at 62.4% and 72.6%, respectively. One of the LN features most often considered in the literature to predict neoplastic invasion is SA diameter. *Saito et al.* found SA diameter superior to LA diameter in predicting metastatic LNs [22]. Based on their analysis of the ROC curves with Youden’s index, a 9 mm cutoff for SA diameter emerged as optimal for detecting metastatic LNs, whereas we found a cutoff of 6 mm for the SA of the largest LN. The overall sensitivity of the former cutoff was lower (55.3% vs. 61.3%), and the specificity was higher (86% vs. 65.6%) than in our study, possibly due to a higher proportion of patients with a high N stage in our sample. *Hasegawa et al.* identified a SA diameter of 8 mm as the optimal cutoff for detecting a metastatic LN [33]. Using the proposed SA cut off for the largest LN, they obtained a lower sensitivity (46.4% vs. 61.3%), and a higher specificity for nodal metastasis (96.8% vs. 65.6%) than in our study. This may be explained by the slice thickness of 7 mm used by *Hasegawa et al.* in their CT scans, as opposed to the 3 mm slice thickness adopted in our study. *Kubota et al.* using a cut-off for the SA of 12 mm obtained an accuracy of 89.6% in the identification of nodal group metastases [35]. *Wang et al.* found SA diameter of LNs superior to LA diameter or the SA/LA ratio for identifying metastatic LNs [32]; and the difference in the AUCs between the former parameter and the latter two was statistically significant (*p* < 0.001). This is consistent with our data, though we identified the sum of the SAs as having the best AUC (0.721; 95%CI: 0.677–0.762). The difference was statistically significant compared with the SA (*p* = 0.0192), the SA/LA ratio of the largest LN (*p* < 0.0001), or the mean of the SA/LA ratios of all the LNs (*p* < 0.0001). In our study, it was the sum of the SAs, rather than the SA diameter, with a cutoff of >8 mm that showed the best sensitivity and specificity, at 62.4% and 72.6%, respectively. Moreover, although statistically significant, the difference between the median of the SAs of the largest LNs in the metastatic vs. non-metastatic nodal group was only of 1 mm. Using a patient-based approach, the sum of the SAs of all the LNs in all the nodal groups confirmed the best AUC on ROC analysis (0.787; 95%CI: 0.697–0.860). Only a few studies considered the sum of the diameters of LNs. *You et al.* demonstrated the correlation between the sum of the SA diameters of LNs and the number of metastatic LNs in GC [21], and we confirmed their finding. 

CT is not the only tool suitable for detecting disease spreading to the LNs. Several studies comparing the accuracy of CT and EUS in detecting metastatic LNs in GC demonstrated their comparable accuracy [30,36], though EUS showed a greater variability in sensitivity and specificity (from 16.7% to 96.8% [median, 70.8%] and from 48.4% to 100% [median, 84.6%], respectively) [17,18]. EUS has the benefit that the transducer can be placed near the lesion to better reveal the involvement of perilesional LNs, but it cannot provide information about any invasion of LNs far from the gastric walls. EUS has some other limitations also [17]. First, its accuracy is operator-dependent. Second, it may require sedation and the procedure may cause complications, morbidity and mortality. Last, and in daily clinical practice, especially at small centers, professionals specialized in EUS are not always available [17]. CT for the preoperative staging of patients with GC is not invasive, more widely available, and its results are reproducible. 

Finally, PET/CT is a modality of choice for systemic staging of gastric cancer, despite detailed studies of accuracy of PET/CT for nodal metastasis identification in GC are lacking. A recent investigation records sensitivity/specificity of 71%/94% of FDG-PET/CT for nodal metastases in a small group of 15 patients [37]. 

Several limitations of our study need to be acknowledged. For a start, this was a single-center retrospective analysis. Only 107 patients with a preoperative CT scan were considered, while patients whose CT scans were performed at other centers, or who underwent urgent treatment with no staging CT scan were excluded. The study cohort also covered a broad timespan (though the treatment of GC has changed very little in the last 5 years). In future, a large multicenter cohort study or prospective study with a standardized protocol would be needed to overcome these limitations and to establish reliable volumetric ranges and sums of SA ranges for loco-regional nodal stations in GC. Secondly, measuring every single LN detected at preoperative CT is too time-consuming to be feasible at every center in everyday clinical practice and artificial intelligence could be helpful in this field, since different radiomics models, both in CT and PET/CT images analysis, proved to be effective in prediction of nodal metastases in GC [38,39,40,41]. Nonetheless, in this study we considered easily-measurable parameters that do not demand specific training for radiologists. As node-by-node matching on histopathology and imaging was unfeasible, we took for reference the presence of metastases in each loco-regional nodal group. Finally, our dataset included a limited set of 18 patients (17%) who had received neoadjuvant treatment, which might have added a bias.

## 5. Conclusions

From the results of our study, the sum of the SAs of the LNs in a nodal group and in all nodal groups emerged as the best predictors of solitary nodal group metastasis and locoregional node metastasis, respectively, in patients with GC. 

## Figures and Tables

**Table 1:** Patients’ demographic, clinical, and treatment characteristics.

Variables		N (% or IQR)
Age	Years, Median (IQR)	72 (62–78)
Gender	Female	40 (37.4)
	Male	67 (62.6)
Preoperative CEA level	(>4 ug/L)	9 (8.4)
BMI	kg/m^2^, Median (IQR)	24.0 (22.4–27.2)
Tumor size	mm, Median (IQR)	40 (25–60)
Tumor site	Cardia (Siewert III type)	8 (7.4)
	Fundus	11 (10.3)
	Body	34 (31.8)
	Antrum	48 (44.9)
	Pylorus	1 (0.9)
	Multicentric disease	2 (1.9)
	Gastric remnant	3 (2.8)
Type of surgical resection	Subtotal gastrectomy	44 (41.1)
	Total gastrectomy	44 (41.1)
	Extended total gastrectomy	11 (10.3)
	Remnant gastrectomy	3 (2.8)
	Proximal gastrectomy	0 (0)
	Esophageal resection	3 (2.8)
	Multivisceral resections	1 (0.9)
Extent of lymphadenectomy	D1	31 (29.2)
	D2	76 (71.7)
Preoperative treatment	Chemotherapy	15 (14.0)
	Radiotherapy	3 (2.8)
Adjuvant treatment	Chemotherapy	67 (62.6)
	Radiotherapy	8 (7.5)
Length of hospital stay in days, median (IQR)		11 (10–13)
Postoperative complications		48 (44.9)
Clavien–Dindo classification	grade 0–2	94 (87.9)
	grade 3–5	13 (12.1)
Deep abdominal collections		18 (16.8)
Bleeding requiring transfusions		15 (14.0)
Anastomotic leakage		5 (4.7)

IQR interquartile range, CEA carcinoembryonic antigen, NOS not otherwise specified, SRC signet ring cell, TNLE total number of nodes examined, LNR lymph node ratio.

**Table 2:** Histopathological characteristics.

Variables		N (% or IQR)
Histotype	Tubular	42 (39.3)
	Poorly cohesive (NOS or SRC)	39 (36.4)
	Other types	26 (24.3)
Lauren’s classification	Mixed	2 (1.9)
	Intestinal	43 (40.2)
	Diffuse	48 (44.9)
	NA	12 (11.2)
Histological grade (n = 144)	G1–G2	33 (30.8)
	G3	53 (49.5)
	NA	21 (19.6)
T stage	T1	18 (16.8)
	T2	12 (11.2)
	T3	32 (29.9)
	T4	45 (42.1)
N stage	N0	38 (35.5)
	N1	14 (13.1)
	N2	14 (13.1)
	N3a	21 (19.6)
	N3b	20 (18.7)
M stage	M1	15 (14.0)
TNM stage	Stage I	24 (22.4)
	Stage II	25 (23.4)
	Stage III	43 (40.2)
	Stage IV	15 (14.0)
Total number of nodes examined	median (IQR)	30 (22.5–44)
Lymph node ratio	median (IQR)	0.12 (0–0.47)
Lymphatic invasion		75 (70.1)
	NA	6 (5.6)
Vascular invasion		47 (43.9)
	NA	6 (5.6)
Radicality	R0	88 (82.2)
	R1	19 (17.8)

IQR interquartile range, NOS not otherwise specified, SRC signet ring cell, NA not available.

**Table 3:** Comparison of lymph node measurements on CT between metastatic and nonmetastatic nodal groups.

Parameters	Metastatic Nodal Groups	Nonmetastatic Nodal Groups	*p* Value *
Short axis of the largest lymph node in the nodal group	7 mm (IQR: 6–9 mm)	6 mm (IQR: 5–7 mm)	<0.0001
Ratio of the short axis to the long axis of the largest lymph node in the nodal group	0.78 (IQR: 0.67–0.88)	0.78 (IQR: 0.67–0.87)	0.8926
Mean of the ratios of the short axis to the long axis of all lymph nodes in the nodal group	0.80 (IQR: 0.71–0.88)	0.78 (IQR: 0.67–0.86)	0.0969
Sum of the short axes of all the lymph nodes in the nodal group	11 mm (IQR: 7–17 mm)	6 mm (IQR: 5–10 mm)	<0.0001
Volume of the largest lymph node in the nodal group	280.8 mm^3^ (IQR: 174.7–624.0 mm^3^)	195.8 mm^3^ (IQR: 130.0–305.7 mm^3^)	<0.0001

* comparison with Mann-Whitney test; IQR inter-quartile range.

## Data Availability

The datasets used and/or analyzed during the current study are available from the corresponding author on reasonable request.
